# Does FANCA Assist CENP-E in Architectural Organization of Chromosomes at Spindle Equator?

**DOI:** 10.4274/balkanmedj.2018.0709

**Published:** 2018-09-21

**Authors:** Amr Ahmed El-Arabey, Salama Abdu Salama, Adel Rashad Abd-Allah

**Affiliations:** 1 Department of Pharmacology and Toxicology, Al-Azhar University Faculty of Pharmacy, Nasr City, Cairo, Egypt

To the Editor,

Fanconi anemia is a complex genetic disorder caused due to a mutation in one of at least 21 Fanconi anemia genes and characterized by developmental abnormalities, congenital malformation, genomic instability, and predisposition to cancer. Indeed, patients with mutation in Fanconi anemia genes, including complementation group A (FANCA), are susceptible to cancer, particularly acute myeloid leukemia and squamous cell carcinoma. Furthermore, Fanconi anemia is associated with different diseases, including congenital abnormalities that may affect all organ systems (1,2). Recently, a novel originator for FANCA mutation has been identified in Romani patients living in the Balkan region (2). The Fanconi anemia proteins play key roles in ensuring proficient DNA damage repair, overcoming replication stress, orchestration of DNA replication, and fine-tuning mitotic checkpoints to ensure faultless chromosome segregation during cell replication. Moreover, FANCA has been implicated in the repair of interstrand DNA crosslinks (3). The study by Du et al. (4) in 2009 demonstrated that FANCA interacts with the C-terminus of the centromere-associated protein E (CENP-E) *in vitro* and *in vivo*. This interaction might suggest a critical role in the mitotic checkpoint signaling pathway. Interestingly, CENP-E is involved in the initial alignment of chromosomes at the spindle equator on the metaphase plate, and therefore, it is required for steady spindle microtubule capture at the kinetochores, which is a critical step in proper chromosome congression during prometaphase. Basically, siRNA-mediated CENP-E silencing results in the failure of chromosome congression to the equator, which is characterized by the clustering of chromosomes near the poles (5,6). Similarly, a recent study demonstrated that FANCA-null cells are associated with defects in chromosome congression. Indeed, FANCA ensures interphase and mitosis over hematopoiesis *in vivo* (7). Furthermore, another study revealed that impairment of spindle assembly checkpoint *in vivo* gives rise to lagging chromosomes, which is an obvious mitotic error in the hematopoiesis of FANCA^−/−^ patients (8). Here we would like to shed light on the strong positive correlation between CENP-E and FANCA co-expression reported in 26 studies from The Cancer Genome Atlas data of different types of cancers (Table 1). Furthermore, statistical analysis of mutual exclusivity and co-occurrence of CENP-E and FANCA in 9377 tissue samples from the previously mentioned studies (Table 1) using cBioPortal Cancer Genomics analysis (http://www.cbioportal.org) demonstrated a significant positive correlation (p<0.001) (Table 2). However, the precise regulatory function of FANCA in ensuring chromosome integrity during mitosis in dividing cells has not yet been elucidated. Collectively, from the previous data, it is clear that FANCA binds to CENP-E and assists in chromosome organization at the spindle equator to ensure timely appropriate chromosome separation (3). Moreover, the current data will open potential new avenue toward the identification of the mechanistic role of FANCA as a DNA repair protein in chromosomal alignment during mitosis.

## Figures and Tables

**Table 1:**
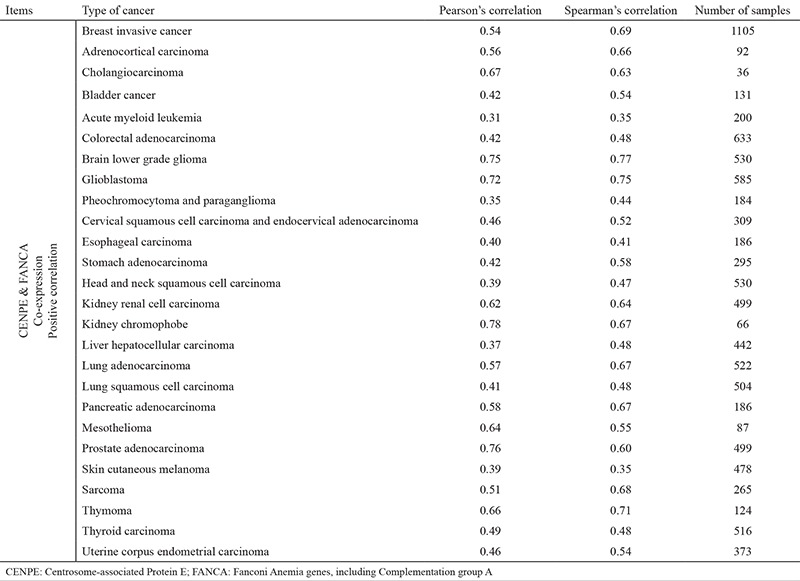
Positive correlation between C-terminus of the Centromere-associated Protein E and FA genes, including Complementation group A in different types of cancers from The Cancer Genome Atlas data

**Table 2:**
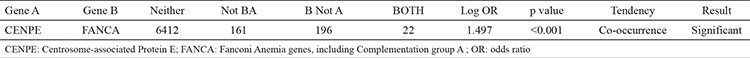
Mutual exclusivity and co-occurrence analysis of C-terminus of the centromere-associated protein E and FA genes, including complementation group A from 26 studies of The Cancer Genome Atlas data (total samples: 9377)
